# A New Approach to Noninvasive-Prolonged Fatigue Identification Based on Surface EMG Time-Frequency and Wavelet Features

**DOI:** 10.1155/2023/1951165

**Published:** 2023-01-30

**Authors:** Fauzani N. Jamaluddin, Fatimah Ibrahim, Siti A. Ahmad

**Affiliations:** ^1^Center for Innovation in Medical Engineering, Faculty of Engineering, Universiti Malaya, Kuala Lumpur 50603, Malaysia; ^2^Department of Biomedical Engineering, Faculty of Engineering, Universiti Malaya, Kuala Lumpur 50603, Malaysia; ^3^Center for Printable Electronics, Universiti Malaya, Kuala Lumpur 50603, Malaysia; ^4^Department of Electrical and Electronics Engineering, Faculty of Engineering, Universiti Putra Malaysia, Serdang 43400, Selangor, Malaysia; ^5^Malaysian Research Institute on Ageing, Universiti Putra Malaysia, Serdang 43400, Selangor, Malaysia

## Abstract

In sports, fatigue management is vital as adequate rest builds strength and enhances performance, whereas inadequate rest exposes the body to prolonged fatigue (PF) or also known as overtraining. This paper presents PF identification and classification based on surface electromyography (EMG) signals. An experiment was performed on twenty participants to investigate the behaviour of surface EMG during the inception of PF. PF symptoms were induced in accord with a five-day Bruce Protocol treadmill test on four lower extremity muscles: the biceps femoris (BF), rectus femoris (RF), vastus medialis (VM), and vastus lateralis (VL). The results demonstrate that the experiment successfully induces soreness, unexplained lethargy, and performance decrement and also indicate that the progression of PF can be observed based on changes in frequency features (Δ*F*_med_ and Δ*F*_mean_) and time features (ΔRMS and ΔMAV) of surface EMG. This study also demonstrates the ability of wavelet index features in PF identification. Using a naïve Bayes (NB) classifier exhibits the highest accuracy based on time and frequency features with 98% in distinguishing PF on RF, 94% on BF, 9% on VL, and 97% on VM. Thus, this study has positively indicated that surface EMG can be used in identifying the inception of PF. The implication of the findings is significant in sports to prevent a greater risk of PF.

## 1. Introduction

Surface electromyography (sEMG) is an electrical field of human skeletal musculature [1]. It is acquired by placing electrodes on the skin surface near the human muscle. The frequency and amplitude of the signals represent the behaviour and condition of the muscle's motor unit, conduction velocity, and ionic alteration of the muscle. Fatigue can be determined by the changes in its frequency content and amplitude either during an activity by analyzing every interval time length [2], at the beginning and ending of the activity [3, 4], or before and after the activity [5–8].

In fatigue detection, frequency shifting represents the changes in muscle fibre conduction velocities and subsequent changes in the duration of the motor unit action potential waveform and fluctuations of muscle force and muscle fibre types as well as their decomposition [8, 9]. Most of the opinions agree that fatigue can be identified when its frequency shifts to a lower value to indicate that the muscle conduction velocities are slowing down [10, 11]. Other than frequency, fatigue can be detected through the amplitude of sEMG signals. The changes in the sEMG amplitude depend on the number of active motor units [12], discharge or firing rates, and the shape and propagation velocity of the intracellular action potential [10]. The amplitude of sEMG tends to increase during submaximal voluntary contraction (during motor unit recruitment) and decrease during maximal voluntary contraction [10, 13–16].

Other than time and frequency features, a new time-frequency feature representation to track fatigue was introduced and is known as the wavelet index (WI) method [17]. WI was introduced since it is more suitable to deal with nonstationary signals such as sEMG [17]. There are five WI features introduced by Malanda and Izquierdo, including the wavelet index ratio between moment −1 at scale 5 and moment 5 at scale 1 (WIRM1551), wavelet index of the ratio between moment −1 at the maximum energy scale and moment 5 at scale 1 (WIRM1M51), wavelet index of the ratio between moment −1 at scale 5 and moment 2 at scale 2 (WIRM1522), wavelet index of the ratio of energies at scale 5 and 1 (WIRE51), and wavelet index ratio between square waveform lengths at different scales (WIRW51). Through WI, the distribution shifting of sEMG energy can be assessed based on its scale and frequency band of decomposition.

In normal conditions, fatigue usually disappears by itself after a while. Recovering from fatigue indicates that biochemical reactions during sports activity are able to return to a normal level [18]. Under normal fatigue (NF) conditions, most opinions agree that the degree of fatigue begins with an increment in amplitude, followed by unchanged and decreased trends, as well as accompanied by a decrement in frequency centers. WI features tend to increase, indicating the distribution of energy shifting to a lower value under NF conditions.

However, high-intensity training activity will commonly lead to more biochemical reactions such as releasing of stress hormones (cortisol, epinephrine, and prolactin) [19, 20], glycogen depletion [21], and the existence of lactate [22]. Fatigue due to intense training will require a longer recovery period than normal physical activity. It is crucial for improvement and recuperation [23]. During the period, it will enable hormones to return to a normal level [18] and allow physiological adaptation to a cardiovascular and muscular system to provide a higher level of performance [24]. If the training load is imbalanced with an inadequate recovery period, fatigue can be continuous and accumulated. This situation leads to prolonged fatigue (PF). Under this condition, more biochemical or maladaptive hormonal responses may occur [24, 25]. The alteration in biochemicals, which leads to PF, can be signified by reduced performance, lethargy, soreness, insomnia, psychological disturbance, restlessness, hypertension, and increased incidence of injury [21, 26]. It commonly requires several days to a week to recover from [23, 27]. This condition needs to be treated accordingly to avoid a more severe condition, known as chronic fatigue syndrome. A report shows that about 20–60% of athletes, 60% of elite runners, and 33% of nonelite runners experienced chronic fatigue syndrome at least once in their career life [23, 26, 28].

In current practice, PF signs can be assessed invasively or noninvasively. Blood tests are invasive and used to investigate biochemical concentrations associated with PF such as lactate, glycogen depletion, creatine kinase, and iron levels [22, 28]. Meanwhile, muscle biopsies are utilized to evaluate the condition of the injured muscle and ionic concentration in the muscle layer. During the collection of muscle tissue, numbing medicine is required. Although both blood tests and biopsies are reliable and accurate, they cause discomfort and are not suitable for frequent measurement. Furthermore, these methods are time-consuming, need to be analyzed in a laboratory environment, and require full supervision from an expert [29].

Due to the limitation of invasive methods, PF can also be traced through noninvasive diagnostic tools because the alteration in biochemicals can be observed physically. For example, glycogen depletion and lactate accumulation are commonly associated with a decline in performance, the oxidative stress increment leads to muscle pain, and cytokine leads to unexplained lethargy, decreased appetite, depression, and sleep disturbance [26, 28]. The commonly used noninvasive tools are interviews, athlete-coach monitoring approaches [18], questionnaires [26, 28], training logs [30], and perceived exertion ratings. The current practice requires more than one diagnostic tool to comprehensively screen off PF signs. Diagnostic tools such as interviews, training logs, and questionnaires often require close supervision by the practitioner and personal coach. Nevertheless, using many tools for the PF identification process is ineffective, particularly, in monitoring a large group of athletes because these tools are time-consuming and have many procedures. Even so, many agree that PF condition prevention is the best solution [26]. The reason is that the treatment of PF is time-consuming and cost-ineffective, depending on the degree of PF. Furthermore, PF signs endured are too risky for athletes.

Later, findings reveal that the center frequency shifting of sEMG to the upper value was attributed to the alteration of ionic concentrations such as lactate and glycogen and the existence of soreness following high-intensity exercise [6, 31]. This finding is opposed to the earlier findings that state a decrement in the center frequency of sEMG following short duration and light exercise refers to fatigue conditions. This situation demonstrates that duration, the intensity of exercise, biochemical reactions, and the existence of PF signs may affect the sEMG signal behaviour. This situation also demonstrates the potential of sEMG as a new tool which is noninvasive, comfortable, fast, easy to use, and quantifiable to detect signs of PF. The detection at the earliest stage helps prevent a more serious state of PF.

Therefore, this paper aims to investigate the ability of sEMG signals to identify the inception of PF in four muscles with different percentages of muscle activation. This paper also investigates the ability of wavelet index features in PF identification. The performance of the surface EMG features was evaluated by the naïve Bayes classification accuracy in predicting the PF condition.

## 2. Materials and Methods

### 2.1. Study Protocol

Twenty participants (age ± standard deviation (SD): 24 ± 3 years old; body mass index: 22.7 ± 2 kg/m^2^) were recruited for this study. Participants were screened using a Physical Activeness Questionnaire (PAR-Q and You) (Supplementary Appendix S1) to rule out any pre-existing health contraindications and risk factors for exercise. The exclusion criteria were participants with diabetes, high blood pressure, heart disease, any chronic disease, joint or bone problems, and taking any medication to control blood pressure and blood sugar. The approval, to conduct the experiment procedure, was obtained from the Ethical Committee, Universiti Putra Malaysia (UPM/TNCPI/RMC/1.4.18.1(JKEUPM)/F2).

The participants were given a written and verbal explanation, including the potential risks and discomfort that they might experience. The participants signed written informed consent before the experiment began. As a precaution, the participants were also protected by insurance (policy number: P809067176) during the whole experiment period.

### 2.2. Procedure

The experimental design was divided into two phases: Phase I was meant for familiarization and Phase II was for intensive training. Each participant had to take part in both phases. Phase I enabled the participants to familiarize themselves with the equipment and procedures, while Phase II was designed to induce PF signs. Between Phase I and Phase II, the participants were requested to rest and refrain from exercising or doing any heavy physical activity. Phase I was carried out on alternate days to avoid the emergence of PF, and Phase II was carried out on five consecutive days (see Supplementary Table S1. Schedule of Experiment).

The participants were instructed to refrain from any heavy physical exercise, alcohol, and caffeine consumption 24 hours before the running test in both phases. They also required taking meals two hours before the assessment to avoid lack of energy and dehydration. The experiment was conducted in accordance with the Bruce Protocol treadmill test (see Supplementary Table S2). In the protocol, the inclination and speed of the treadmill were increased every three minutes. The total duration of the protocol was 21 minutes. The participants were required to run for five consecutive days and requested to improve their performance daily. As individual fatigue response is highly variable, no specific distance and time duration were fixed [32].

### 2.3. Data Collection

In this study, training logs (see Supplementary Appendix S2) were used to record measurements before, during, and after the running activity. The measurements were used to monitor the daily performance and identify the emergence of PF conditions during Phase II of the experiment. The flowchart of the experiment procedure and measurements is shown in Supplementary Figure S1, and the equipment utilized throughout the experiment was the COSMED T170 treadmill, Polar chest strap heart rate monitor, Watsons blood pressure monitor, and custom-made surface EMG data collection tool (see Supplementary Figures S2(a) and S2(b) for the schematic circuit of surface EMG systems).

The recorded measurements during Phase II were as follows:

(a) Percentage of the maximal heart rate
Percentage of the maximal heart rate (%HR_max_) is recorded to indicate running efforts performed by the participant. %HR_max_ is determined as
  (1) $$\begin{array}{c} \%{HR}_{\max}=\frac{{HR}_{\max}\left(running\right)X\;100}{\left(220-Age\right)}\text{.} \end{array}$$
(b) Percentage of endurance time
Endurance time of running on the treadmill is calculated [33] based on the following equation:
  (2) $$\begin{array}{c} \%T_{endu\;rance}=\frac{T_{recorde\;d}\;X\;100}{21minutes}\text{.} \end{array}$$
(c) Prolonged fatigue sign identification

The participants were also requested to fill in a 24-hour training distress questionnaire (see Supplementary Appendix S3) daily [28]. The questionnaire was used to identify sleeping and psychological disturbance and muscle soreness. During the experiment, the participants were also interviewed before the running activity. The interview session was conducted to identify whether the participants experienced lethargy. After running, the participant requested to scale the running activity experiment to indicate the difficulty of the experiment. The emergence of PF signs was monitored based on noninvasive diagnostic tools, as summarized in Table 1.

The prolonged fatigue diagnosis was important as surface EMG signals were then grouped and classified based on PF signs experienced by the participants. Due to ethical reasons and potential risks endured by the participants, only symptoms developed within five days of the experiment were monitored. The earliest PF signs that appeared during the training were sufficient to indicate the emergence of PF. The participants were also reminded about two symptoms of fatigue. The symptoms were observed based on two conditions as follows:

(a) Fatigue symptom 1 (monitored before the running activity)
The participant was not allowed to run and was terminated from the experiment if any of the following fatigue symptoms were observed before the assessment:
  - Heart rate >100 bpm
  - Blood pressure >140/90
  - Showing performance decrements in the previous experiment
  - Psychology scores in the 24-hour training distress questionnaire >14 for at least three days
  - Collapsing in the previous experiment
  - Fatigue symptom 2 (monitored during running activity)
  - The participant must stop running if the following symptoms are observed while running:
  - Lack of energy
  - Feel dizzy
  - Blurred vision

### 2.4. Surface Electromyography

**Figure 1:**
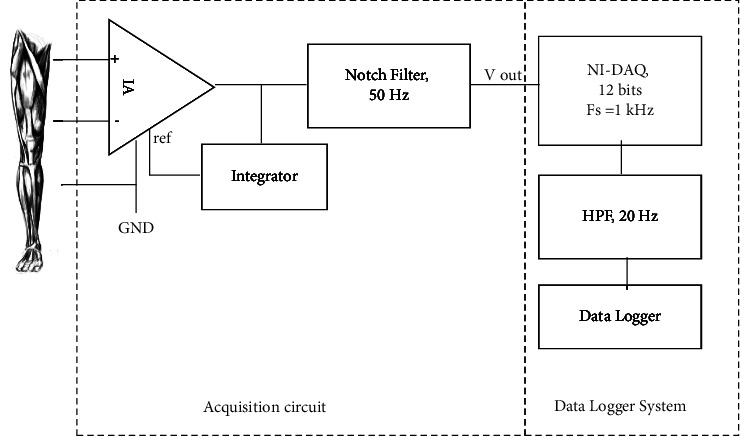
Block diagram of the sEMG data acquisition system.

SEMG signals were collected from the biceps femoris (BF), rectus femoris (RF), vastus lateralis (VL), and vastus medialis (VM). These muscles were selected based on the activation muscles during running and suffer a high rate of injury in sports involving running [35, 36]. Running at 10° grade inclination activates 79 ± 7% of BF, 76 ± 14% of vastus, and 44 ± 20% of RF, and the activation is elevated as the inclination increases [36, 37]. The sEMG signals were collected using the custom-built sEMG acquisition system, as shown in Figure 1.

The sEMG system was designed based on an AD620 instrumentation amplifier system. AD620 was selected as it provides a 130 dB common-mode rejection ratio (CMRR), low power consumption, that is, 1.3 mA, and comprises a low input voltage noise of 9 nV/√Hz at 1 kHz and 0.28 *μ*V p-p in the 0.1 Hz–10 Hz band. The full system offers a signal-to-noise ratio (SNR) of 25 dB and gains an amplifier at 248. The 50 Hz notch filter is designed according to the following equation:

(3) $$\begin{array}{c} F_{n}=\frac{1}{2\pi RC}\text{,} \end{array}$$

where *R* = 68 k Ω and *C* = 47 nF.

The schematic diagram of the sEMG data acquisition board is depicted in Supplementary Figures S2(a) and S2(b). The analog signals of surface EMG were digitized into 12 bits by using National Instrument Data Acquisition (NI-DAQ) 6008 with frequency sampling, *F*_*s*_ at 1k Hz. 1k Hz was selected to avoid aliasing as suggested by De Luca. Then, the collected data were filtered using a digital finite impulse response (FIR), a high-pass filter (HPF), 301 taps, and a cutoff at 20 Hz. This HPF is essential for removing baseline wander during data acquisition. *F*_*s*_ and HPF specifications were set using data logger software, LabVIEW.

Ag/Ag Cl electrodes from Kendall MediTrace 200 were used to acquire the signals. Bipolar electrodes with 20 mm inner distance were attached to the involved muscles, and one reference electrode was placed at the knee of the participants. The electrodes were positioned at BF, RF, VL, and VM based on the Surface EMG for the Noninvasive Assessment of Muscles (SENIAM) standard [38]. The RF muscle was determined by 50% distance between the patella upper borders and the anterior iliac spine (AIS), VL was at 25% distance from Gerdy prominence to AIS, and VM was at 25% distance from the joint space to AIS. After measuring and marking the muscle, palpation of the involved muscle was carried out to ensure that the electrodes were placed correctly. During palpation, the participants were asked to flex and extend knee movements to activate the muscles, as shown in Supplementary Table S3 [38, 39].

**Figure 2:**
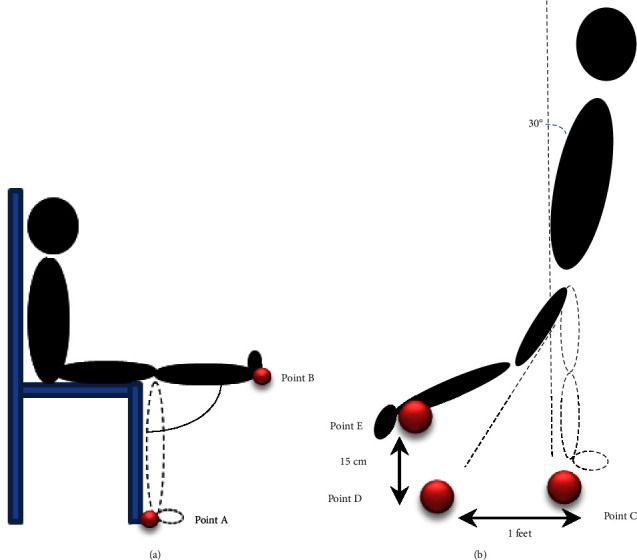
Leg movement to activate (a) RF, VL, and VM muscles and (b) BF.

In data collection, the participants were asked to move their legs to activate the observed muscles. Only one leg was involved in data collection, and it was observed that the participants were comfortable using the left leg in the study. The investigation on one leg was enough in this study to observe PF conditions based on surface EMG. RF, VL, and VL were activated when the hip was flexed, while the knee was extended to 180. As shown in Figure 2(a), the participants were asked to sit on a chair and were requested to move their legs from Point A to Point B to activate the quadriceps muscle group. They were asked to stay at each point for ten seconds and then repeat the movement three times. It was discovered that the RF, VL, and VM muscles contracted when the leg was at Point B and were at rest when the leg was at point A, as suggested by Konrad.

**Figure 3:**
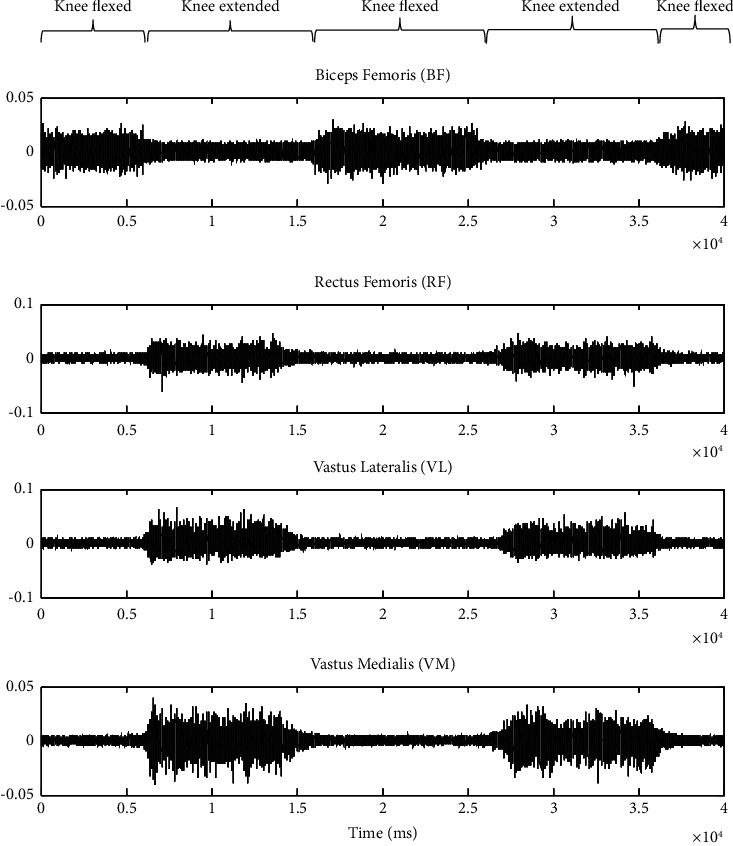
Example of sEMG signals collected from BF, RF, VL, and VM.

Other than RF, VL, and VM, surface EMG signals were also collected from the BF muscle. The location of the electrodes for BF was at 50% distance from the lateral epicondyle of the tibia to the ischial tuberosity. To collect surface EMG signals from BF, the participants were asked to stand and move their legs from Point D to Point E, as shown in Figure 2(b). Before that, the participants needed to place one (1) of their legs one foot (1 ft) away from Point C, which was Point D. BF contracted when the hip was extended, while the knee flexed [34]. When the body gesture was about 30 forward, the knee flexed until the leg was lifted about 15 cm to Point E. This distance was chosen as it provides the maximum activation of BF during movement [40]. The participants were requested to move their legs from Point D to Point E three times at (10) seconds intervals. The sEMG signals were collected during before and after running activities.  Figure 3 shows the example of sEMG signals when the knee is flexed and extended during the position and movement in Figures 2(a) and 2(b).

### 2.5. Feature Extraction

**Figure 4:**
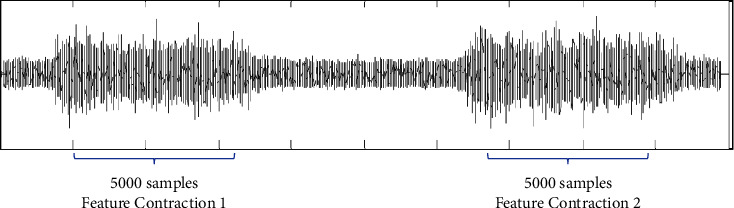
Nonoverlapping windowing technique in feature extraction.

A nonoverlapping windowing technique was employed with samples *n* = 5000, as shown in Figure 4. The moment at which the muscles started to contract and relax was ignored because the dynamic movement during data collection might result in false information [41]. The number of *n* was selected because the authors of [2] have demonstrated that segmentation length is suitable for muscle fatigue identification. The features were extracted at each contraction and averaged.

Specific features were extracted based on the frequency, time, and wavelet index (WI). The spectral content of surface EMG was determined according to the Fourier transform, and the frequency parameter was quantified based on its median (*F*_med_) in (4) and mean (*F*_mean_) in (5):

(4) $$\begin{array}{c} Fmed=\frac{1}{2}\;\overset{M}{\underset{j=1}{\sum }}P_{j}\text{,} \end{array}$$

(5) $$\begin{array}{c} Fmean=\frac{\overset{M}{\underset{j=1}{\sum }}f_{j}P_{j}}{\overset{M}{\underset{j=1}{\sum }}P_{j}}\text{,} \end{array}$$

where *f*_*j*_ = frequency of the spectrum at frequency bin *j*, *P*_*j*_ = EMG power spectrum at frequency bin *j*, and *M* = length of the frequency bin.

The time features can be quantified based on the mean absolute value (MAV) in (6) and the root mean square (RMS) [42] in (7) [42]:

(6) $$\begin{array}{c} MAV=\frac{1}{n}\overset{n}{\underset{j=1}{\sum }}\left|x_{j}\right|\text{,} \end{array}$$

(7) $$\begin{array}{c} RMS=\sqrt{\frac{1}{n}\overset{n}{\underset{j=1}{\sum }}{x_{j}}^{2}}\text{,} \end{array}$$

where *x* = signals and *n* = number of samples [43].

**Figure 5:**
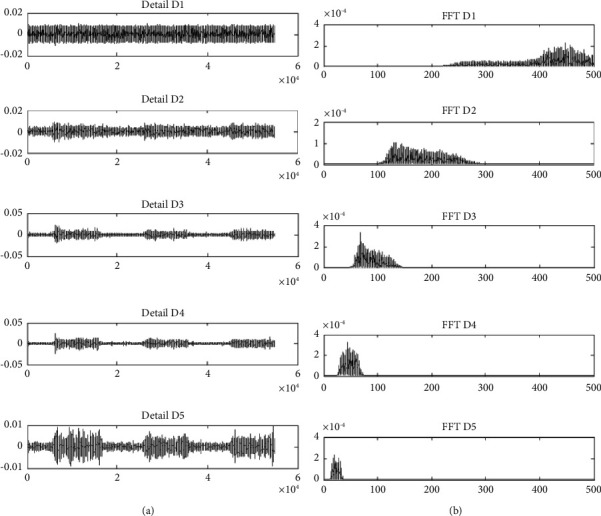
(a) sEMG wavelet details at scales 1–5. (b) Power spectra using the Fourier transform of wavelet details at scales 1–5.

This study also investigates the ability of WI features in PF [43] identification since it was never tested in determining fatigue under high-intensity conditions. WI features were used to evaluate the distribution shifting of sEMG energy based on its scale and frequency band decomposition. WI was calculated based on the discrete wavelet transform (DWT) which was decomposed into five levels by using symlet 5 (sym5) and Daubechies (db5) as the mother wavelet [17]. The decomposition process consisted of a series of filter banks, where at every *i* level of decomposition, the signal was filtered into half of the frequency band [44]. The low-pass filter produced an approximation coefficient, while the high-pass filter produced a detail coefficient (*D*_*i*_) (scales).  Figure 5 shows the five levels of sEMG decomposition details and the power spectra of decomposition details at scales 1–5 that are determined based on the Fourier transform.

The wavelet index ratios between moments at different scales were then determined based on the power spectrum of wavelet details, *D*_*i.*_.

The five WI features were tested as follows:

(a) The WI ratio is between moment −1 at scale 5 and moment 5 at scale 1 (WIRM1551).
  (8) $$\begin{array}{c} WIRM1551=\frac{\int_{f1}^{f2}f^{-1}D_{5}\left(f\right).\;df}{\int_{f1}^{f2}f^{5}D_{1}\left(f\right).\;df}\text{,} \end{array}$$
where *ψ*(*t*) used was sym5, *f*1 = 10 Hz and *f*2 = 500 Hz, and *D*_5_(*f*) and *D*_1_(*f*) are the power spectra of the five and first scales of decomposition details [14].
(b) The WI ratio is between moment −1 at the maximum energy scale and moment 5 at scale 1 (WIRM1M51).
  (9) $$\begin{array}{c} WIRM1M51=\frac{\int_{f1}^{f2}f^{-1}D_{\max}\left(f\right).\;df}{\int_{f1}^{f2}f^{2}D_{2}\left(f\right).\;df}\text{,} \end{array}$$
where *ψ*(*t*) used was db5, *f*_1_ = 10 Hz and *f*_2_ = 500 Hz, and *D*_max_ in this work was scale 4 [14].
(c) The WI ratio is between moment −1 at scale 5 and moment 2 at scale 2 (WIRM1522).
  (10) $$\begin{array}{c} WIRM1522=\frac{\int_{f1}^{f2}D_{5}\left(f\right).\;df}{\int_{f1}^{f2}f^{2}D_{2}\left(f\right).\;df}\text{,} \end{array}$$
where *ψ*(*t*) used was db5 and *f*_1_ = 10 Hz and *f*_2_ = 500 Hz. [14]
(d) The WI ratio of energy at scales 5 and 1 (WIRE51) is
  (11) $$\begin{array}{c} WIRE51=\frac{\overset{N}{\underset{j=1}{\sum }}D_{5}^{2}\left[n\right]}{\sum_{j=1}^{N}D_{1}^{2}\left[n\right]}\text{,} \end{array}$$
where *ψ*(*t*) used was sym5 [14].
(e) The WI ratio is between square waveform lengths at different scales (WIRW51).
  (12) $$\begin{array}{c} WIRW51=\frac{\overset{N}{\underset{j=2}{\sum }}\left|{\left.D_{5}\left[j\right]-D_{5}\left[j-1\right]\right|}^{2}\right.}{\overset{N}{\underset{j=2}{\sum }}{\left|D_{1}\left[j\right]-D_{1}\left[j-1\right]\right|}^{2}}\text{,} \end{array}$$
where *ψ*(t) used was sym5 [14].

During the extraction of WI features, frequency sampling *F*_*s*_ = 1k Hz [44] and *n* = 1024 were used. The WI features were then log-transformed to follow the normal distribution.

Fatigue identification always refers to the increment or decrement in the features before and after the activity [45]. The changes and shift of the features (*F*) in this study were quantified by

(13) $$\begin{array}{c} \Delta \;F=Fpost\;–\;Fpre\text{.} \end{array}$$

The positive value of Δ*F* indicates a feature increment for postexercise, whereas the negative value indicates feature decrements.

### 2.6. Statistical Analysis

The features of BF, RF, VL, and VM were preliminarily grouped into two categories: normal fatigue (NF) and prolonged fatigue (PF). They were distinguished based on PF signs explained in Table 1. While the features of the participants who did not experience PF conditions were grouped into NF, the features of the participants who experienced PF conditions were grouped into PF. A *t*-test was conducted, and a significant value was set at *P* < 0.05.

### 2.7. Daily Plot of Surface EMG Behaviour

The daily plot of sEMG behaviour for NF and PF conditions was performed to investigate the progression of fatigue in different muscles with different activation percentages. It was plotted based on Δ*F*_med_ and ΔRMS since these two features commonly represent time and frequency information on surface EMG in fatigue identification. A daily plot was also conducted based on five ΔWI features. To identify the changing behaviour of the features during the emergence of PF, the features were normalized by plotting them from a day before the emergence of PF and the first three days under PF conditions. The reasons were the individual's responses to PF signs that varied, and the normalization will help understand the trend line of sEMG features during the intensive training period specifically under PF conditions.

### 2.8. Classification

The classification process began with selecting features and reducing the dimension of the features. The classification was performed based on different feature selections to investigate the optimum classification performance based on the selection. The feature selections were based on the following features:

- Time features: ΔMAV and ΔRMS
- Frequency features: Δ*F*_med_ and Δ*F*_mean_
- Time and frequency features: ΔMAV, ΔRMS, ΔFmed, and ΔFmean
- Wavelet index features: ΔWIRM1551, ΔWIRM1M51, ΔWIRM1522, ΔWIRE51, and ΔWIRW51
- Time, frequency, and wavelet index features: ΔMAV, ΔRMS, ΔFmed, ΔFmean, ΔWIRM1551, ΔWIRM1M51, ΔWIRM1522, ΔWIRE51, and ΔWIRW51

From the feature selection, dimensionality reduction was employed to reduce the complexity and computation time of the classification algorithm, increase accuracy, and decrease overfitting problems [46]. Data reduction in this work was carried out based on linear discriminant analysis (LDA). This method maximizes the intercluster distance between classes and minimizes the intracluster distance within classes in the transformation of reduced features. In LDA, the original dimensional feature space was transformed into a lower dimensional feature space, without losing any important information [46].

In the classification stage, the naïve Bayes (NB) technique was applied to discriminate NF and PF classes. This method was selected as it was previously utilized in experiments studying fatigue classification [36, 37]. NB is one of the established statistical pattern recognition methods [46]. NB classifier functions are based on the probability distribution of the feature vector, *x*. *x* belongs to class *ω*_*m*_ which is computed from probability distribution conditioned on the class *ω*_*m,*_*P*(*x*|*ω*_*m*_), by assuming class-conditional independence of the features:

(14) $$\begin{array}{c} P\left(x|\omega_{m}\right)=\overset{d}{\underset{k=1}{\prod }}P\left(x^{\left(k\right)}|\omega_{m}\right)\text{,} \end{array}$$

where *d* is a dimension of the feature instance *x*. Equation (13) requires that the *k-*th features of the instance, which is *x*^*(k)*^, are independent of all other features, given the class information.

The probability of the *x* class itself is characterized by

(15) $$\begin{array}{c} P\left(x\right)=\overset{d}{\underset{k=1}{\prod }}P\left(x^{\left(k\right)}\right)\text{.} \end{array}$$

The classification rule was computed from the discriminant function *g*_*m*_(*x*) to represent posterior probabilities as

(16) $$\begin{array}{c} g_{m}\left(x\right)=P\left(\omega_{m}\right)\overset{d}{\underset{k=1}{\prod }}P\left(y^{\left(k\right)}|\omega_{m}\right)\text{.} \end{array}$$

It was represented for each *m*-class. Meanwhile, the *x* class is determined by the largest *g*_*m*_(*x*) computation.

In this work, *k*-fold cross-validation (CV) was adopted for training the classifier. The performance of the classification was evaluated for the accuracy, specificity, precision, and average CV error (CVErr).

## 3. Results and Discussion

### 3.1. Physiological Measurements

Table 2 shows a daily %HRmax record. It indicates that about 18 participants ran at their maximal effort by showing %HRmax >80%, based on the Edwards Intensity Zone 1992. Running at this rate caused the participants to experience heavy breathing and muscular fatigue. It proves that the Bruce Protocol treadmill test provides the high training intensity required in this experiment. High-intensity exercise is essential for inducing faster PF signs. Physiological fatigue responses under PF conditions are tabulated in Table 3. It shows that the first PF sign developed was muscle soreness, which was on day 2 (D_2_) of the assessment. This situation was found to be similar to other studies in [6, 47], whereby soreness developed as early as 24 hours after strenuous exercise.

Table 3 also indicates that PF signs accumulated with performance decrement starting at day 4 (D4) of intensive training. The results agree with [16] as the untreated PF condition develops more PF signs. Apart from that, the results suggest that only three PF signs appeared within five days of intensive training including soreness and performance decrement. Moreover, the results reveal that these are the earliest signs of PF developed in the study. The result in Table 3 further shows that none of the participants experienced psychological and sleeping disturbance, restlessness, and hypertension following intensive training. Hence, the classification of collected surface EMG signal features was based on physiological responses identified in Table 3. The term PF condition afterward refers to muscle soreness, performance decrement, and lethargy.

### 3.2. Surface Electromyography

**Figure 6:**
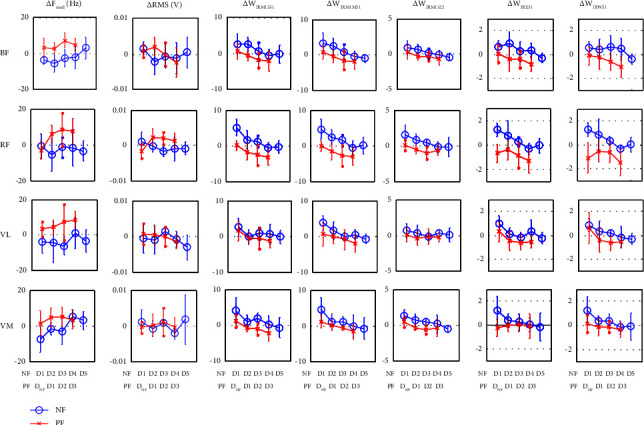
Daily plot of changes in muscle features under NF and PF conditions.

The daily plot of sEMG feature behaviour is displayed in Figure 6, while the bar plot represents standard deviation, “*o*,” and “*x*,” symbols represent the mean value of features in NF and PF conditions, respectively. The features under NF conditions were plotted from day 1 to day 5 (D_1_–D_5_) of the assessment, whereas the plots under PF conditions were normalized from the day before the emergence of PF (D_NF_) to the first three days of the occurrence of PF signs (D_1_–D_3_).

#### 3.2.1. Frequency Feature

Theoretically, the frequency information changes in sEMG describe the behaviour of conduction velocities inside the muscle and subsequent changes in the duration of the motor unit action potential waveform and fluctuation of muscle force and muscle fibre types as well as their decomposition [8, 9]. The frequency spectrum shift information is represented by its mean (*F*_mean_) and median (*F*_med_) in assessing muscle fatigue [42].

Figure 6 shows that Δ*F*_med_ resulted in a negative value for BF, RF, VL, and VM under NF conditions. The negative values of Δ*F*_med_ demonstrate that *F*_med_ was decreasing postrunning activities. The decrement in *F*_med_ was like the most dominant opinion where frequency tends to shift to a lower value to characterize fatigue. The decrease in the centre of frequency as a result of reduced muscle conduction velocity and a change in the frequency spectrum was brought on by the absence of high threshold motor unit recruitment. However, Δ*F*_med_ shows positive values for BF, VL, and VM on day 4 (D_4_) and day 5 (D_5_) under the NF condition. The positive values of Δ*F*_med_ indicate that the median frequency spectrum was shifted upwards. An increase in *F*_med_ was also identified on the day before PF signs appeared (D_NF_) for the BF, VL, and VM muscles, and this behaviour was sustained throughout the PF condition.

The plot in Figure 6 also indicates that the positive values of Δ*F*_med_ only occurred in RF during PF conditions. Statistical analysis reveals that an increment in Δ*F*_med_ under PF is significant at *P* < 0.05 for BF, RF, VL, and VM, as tabulated in Table 4. An increase in *F*_med_ of sEMG during fatigue was rarely reported. The increasing center of frequency was once reported by [48] during the first 30 minutes of recovery from dynamic exercise at a load of 80% of the VO_2_ max. The increasing center of the frequency was reported following the elevation of temperature and lactate after high-intensity dynamic exercise [48]. The relationship between the skin and muscle temperature and the increasing center of the sEMG frequency spectrum was later confirmed in [49]. The positive linear relationship between the temperature and median frequency might be due to an increase in the muscle conduction velocity to increase the power output [49, 50]. The relationship between the frequency of sEMG and temperature was also discussed in [51].

In [51], the authors demonstrated that there is a less effect of temperature on muscle strength and frequency of sEMG but related other possibilities that affected the frequency features such as different recruitment properties of the motor units and the percent of fats and slow twitch motor units under electrodes. Heavy dynamic exercise might also contribute to the substitution of muscle groups following an effect on the alpha motor neuron pool through reflex inhibition that alters recruitment properties. The effect of the neural drive on the muscle and its motor unit action potential was also identified as one of the factors that affect sEMG components [52].

The neural drive for the muscle factor might be related to fatigue induced in the peripheral and central systems. Fatigue in the central system occurs when neurochemical in the brain is altered and stress hormones are secreted. When this happens, it will modify the peripheral information in the contracting muscles and affect the characteristic of sEMG [53–56].

The increment in frequency information features Δ*F*_med_ under PF conditions also might be due to fatigue at the peripheral system. Fatigue at the peripheral system arises from the muscle itself when there is impairment of the peripheral mechanism due to high-intensity exercise as demonstrated by participants in this experiment [45, 49, 50]. High-intensity exercise reduces blood flow due to intense muscle contraction which causes the inadequacy of oxygen supply to the muscle. This situation is also known as an anaerobic condition [54]. The inability to get enough oxygen triggers a biochemical reaction in allowing muscle contraction [57, 58]. An inadequate recovery period causes the inability of ionic alteration during high-intensity exercise to return to its normal level and continue to accumulate. This situation is signified by the emerging PF signs such as soreness and performance decrement. The ionic changes most probably involve glycogen breakdown and the presence of lactate concentration. It is supported by the recorded %HRmax during the running activity, of which 80% and above commonly involves anaerobic contraction. In anaerobic contraction, glycogen and lactate concentration play important roles in ensuring muscle continuous contraction [57, 58]. Furthermore, the alteration in glycogen stores normally leads to soreness and performance decrement due to inadequate fuel for workload [22, 31], and the release of lactate contributes to fatigue and muscle pain, as experienced by the participants in this study. This situation is supported in [31, 48] that also demonstrated that the alteration of both concentrations led the frequency of sEMG to shift to the upper value.

Figure 6 also demonstrates that there were different increment trends in Δ*F*_med_ among the investigated muscles. The trends that happened might be related to muscle activation during running activity. Running at a higher slope such as in the Bruce Protocol treadmill test requires more muscle activation from BF, VL, and VM than from RF, as demonstrated in [36, 37]. A previous study shows that more muscle activation leads to faster progression of fatigue [59]. This study has demonstrated that changes in Δ*F*_med_ happen faster in more activated muscles than in less activated ones. The fast changes in the frequency made the observation of PF signs through more activated muscles rather difficult. The reason was the frequency feature increased even without the emergence of PF signs. Indirectly, an increase in the median frequency might be due to an increase in the muscle temperature and muscle conduction velocity to increase the power output, substitution of the muscle group and recruitment properties, and alteration of ionic concentration underlying the muscle that progressed faster in muscle activation during running. This study has also proved that PF conditions could be easily observed from less activated muscles such as RF because the increment in frequency only occurred under PF conditions. It indicates that PF can be easily identified when frequency from less activated muscles starts to increase.

#### 3.2.2. Time Features

Muscle activity can be observed through its amplitude during the contraction in time-domain representations. In fatigue identification, changes in its amplitude signify the degree of fatigue experienced by the subjects. As exhibited in Figure 6, ΔRMS of BF, RF, and VM under NF conditions increased on D_1_ and decreased on the following days. This progression is similar to dominant opinions that with an increment in amplitude, the decrement in behaviour in characterizing the degree of fatigue soon follows [10].

However, the ΔRMS increased again, as shown in D5, in the BF and VM muscles (Figure 6). Theoretically, in normal conditions, when the load increases, the amplitude tends to have a larger decrement. In this study, the load refers to the endurance time, for which the participants were asked to improve their performance daily. Based on the plot of Δ*F*_med_ on similar days D_5_ on BF and VM, the median frequency shows an increment. In the previous section, the increments in Δ*F*_med_ were related to an increase in temperature. However, the findings in [48, 49] have shown that the increment in the muscle and skin temperature will reduce the amplitude of sEMG signals. The increment in ΔRMS indicated by the BF and VL muscles in D_5_ might be due to the release of free-resting calcium which resulted in force potentiation and led to the increment in EMG, as demonstrated by [60].

The increment in ΔRMS especially under PF might also be due to the changes in ionic concentration. The changes in the ionic concentration were observed through frequency feature behaviour in the precious section. The findings in [61] reported that there was a curvilinear positive relationship between lactate concentration (after reaching a certain lactate threshold) and the amplitude of sEMG.

Apart from that, Figure 6 discloses that ΔRMS started to decrease again on D_2_ of PF, specifically in BF and VL. The decrement in ΔRMS under PF conditions was discovered in [31, 62]. Both studies have proved that amplitude decreases during the emergence of soreness. Nevertheless, another study reveals that the amplitude increases under similar conditions [63]. Therefore, it is reliable to state that the amplitude increases or decreases under PF conditions. The increment and decrement in amplitude under PF also show the degree of fatigue experienced by the muscle. This is attributed to the decrement in amplitude under PF which occurred in highly activated muscles like BF, VL, and VM. High activation led to the fast progression of fatigue. It began when the frequency features started to increase, followed by the amplitude which also increased. Then, it continued with the decreased behaviour to show a certain degree of fatigue experience. This finding was also supported by the progression of fatigue mapped on RF, which was less activated in the study. Figure 6 demonstrates that the RF muscle under NF conditions for ΔRMS continued to decrease (by showing a negative value) and only increased for ΔRMS under PF conditions. The transition behaviour of ΔRMS in the RF muscle was actually similar to that of the Δ*F*_med_ situation, whereby the shifts (from decreasing to increasing) only occurred under PF conditions. The behaviour of ΔRMS for the RF muscle, which decreased under NF and increased under PF conditions, is statistically significant at *P* < 0.05 (Table 4). The statistical test also indicates that the behaviour of ΔRMS under both conditions for BF, VL, and VM is not significant at *P* < 0.05 due to the fluctuation trend in the daily plot (see Figure 6).

#### 3.2.3. Wavelet Indices

This study also investigates the ability of WI features in PF identification. The five WI features were studied, as proposed by [43]. WI features tended to have similar behaviour and response to BF, RF, VL, and VM, as observed in Figure 6. They also tended to increase under NF conditions and decrease under PF conditions.

Figure 6 for ΔWIRM1551, ΔWIRM1M51, and ΔWIRM1522 illustrates the transition of the increment and decrement in WI features under NF conditions. It is also important to note that the features were constantly decreased under PF conditions. The increment (positive value) in WI features under NF in Figure 6 is similar to the results demonstrated in [17]. The increment in the features specifies that the energy distribution shifted to a lower frequency band indicating similar behaviour of frequency, which tended to decrease to show fatigue conditions [10].

Figure 6 also demonstrates that the increment and decrement transitions occurred faster in high-activated muscles such as BF and VL. These situations can be observed under NF conditions on D_4_ and D_5_ for ΔWIRM1551, ΔWIRM1M51, and ΔWIRM1522. ΔWIRM1551, ΔWIRM1M51, and ΔWIRM1522 features also demonstrate that PF could be easily identified in RF, as it occurred on the Δ*F*_med_ and ΔRMS daily plot. The decrement in ΔWI features was caused by energy distribution which slowly shifted to a higher frequency band, which caused the energy distribution at the lower frequency band of decomposition to decrease.

The WIRE51 feature was quantified in accord with its coefficient details *D* of decomposition. The increment in ΔWIRE51 features showed a higher value of *D* at level 5 postexercise than preexercise. Figure 6 indicates that similar trends also appeared in another daily plot of WI features, of which ΔW_IRE51_ gradually decreased under NF plots. Furthermore, the value constantly decreased under PF conditions. Although *D* indicates the time representation of decomposition, ΔWIRE51 proved that the behaviour of the features did not rapidly fluctuate as demonstrated by ΔRMS behaviour. The robustness and sensitivity of WI in dealing with nonstationary behaviour in sEMG were exhibited.

ΔWIRW51 was used to show accumulated changes in the waveform length ratio at *D* level 5 to *D* level 1. Through waveform length behaviour, the duration, frequency, and amplitude of the surface EMG signals were effectively compressed [17]. The increment in ΔWIRW51 in Figure 6 under NF suggests that the surface EMG waveform fluctuated faster postexercise than preexercise. The features gradually decreased to indicate the fluctuation of the surface EMG waveform at *D* level 5 which was getting slower during postexercise. ΔWIRW51 persistently decreased under PF conditions. Hence, it signifies that, apart from the amplitude and energy distribution in the spectra, the waveform characteristic of surface EMG also changed due to PF.

Although BF, RF, VL, and VM demonstrate similar behaviour of WI features under NF and PF conditions, statistical results indicate that all five ΔWI features are only significant at *P* < 0.05 for the RF muscle, as tabulated in Table 4.

### 3.3. Classification

Table 5 indicates the classification results based on the NB method in identifying PF conditions. This result reveals the ability of sEMG features to distinguish between NF and PF based on the naïve Bayes (NB) classification method. Table 5 tabulates the lowest accuracy results from time features of BF, VL, and VM, due to fast fluctuation and overlapping plots of time feature values displayed in Figure 6. This condition makes predicting PF conditions through these features quite difficult. The results revealed in Table 5 indicate that the frequency features had better classification accuracy than time features. Better accuracy was assisted by the significant statistical test results and daily plots to distinguish between NF and PF of frequency features.

The results in Table 5 reveal that the feature selection based on time and frequency offers high-performance accuracy, specificity, and precision in comparison with other feature selections. Thus, it can be concluded that both the time and frequency features of sEMG are significant for PF identification. In this study, the combination of time and frequency feature selections offers accuracy at a rate of 94% on BF, 98% on RF, 95% on VL, and 98% on VL in distinguishing PF conditions. The classification of performances in Table 5 proves the ability of WI features in PF detection. The result shows that WI features produced good classification accuracy in BF (82%), RF (91%), and VL (80%) and less in VM (66%).

## 4. Conclusions

In conclusion, this study has demonstrated that the presence of PF can be identified using the surface EMG signals. The study also introduced a new quantitative noninvasive method to monitor the progression of fatigue, specifically in the muscle of athletes. This monitoring method can provide information to athletes on their performance, and they can perform at their optimum energy. This noninvasive method is suitable to be applied in the sports field for fatigue management and prevent chronic fatigue syndrome for athletes.

## Figures and Tables

**Table 1:** Prolonged fatigue sign identification.

Tools	Prolonged fatigue signs	Identification
Training log	Performance decrement	Endurance time previous workout better
	Restlessness	HR > 100 before running
	Hypertension	BP > 140/90 before running

24-hour training distress questionnaire [28]	Sleeping disturbance	The different time duration between before and during intensive training
	Psychological disturbance	Psychological score >14
	Muscle soreness	Soreness scale (scale 4: tender but not sore to scale, 7: very sore)

Interview	Unexplained lethargy	Feel lethargy before running

Borg Scale CR10 [34]	The difficulty level of exercise increases	Increasing of the scale

**Table 2:** Intensity of training based on percentage of the maximal heart rate.

Intensity zone %HRmax	Day_1	Day_2	Day_3	Day_4	Day_5
Very hard 90–100	10	8	8	11	10
Hard 80–89	8	9	7	5	4
Moderate 70–79	—	1	3	2	1
Light < 69	2	2	2	2	2
Mean ± SD	86 ± 13	86 ± 12	84 ± 14	85 ± 15	87 ± 14

**Table 3:** Number of participants under prolonged fatigue conditions based on physiological responses.

		Day 1	Day 2	Day 3	Day 4	Day 5
Performance improvement			20	20	17	12
Performance decrement			—	—	3	5
Muscle scale	1 (excellent)	2	1	1	1	1
	2 (very good)	6	5	4	2	3
	3 (good)	12	12	10	11	8
	4 (tender, but not sore)	—	1	3	4	7
	5 (sore)	—	1	2	2	2
Psychology score<14		20	20	20	20	20
No sleeping disturbance		20	20	20	20	20
No lethargy		20	20	17	16	15
Lethargy		—	—	3	4	5
HR before run <100		20	20	20	20	20
BP before run <140/90		20	20	20	20	20

**Table 4:** Statistical analysis on the four muscle features.

Features (mean ± SD)										
Muscles		Δ*F*_mean_	Δ*F*_med_	ΔMAV	ΔRMS	Δ WIRM1551	Δ WIRM1M51	Δ WIRM1522	Δ WIRE51	ΔWIRW51
Biceps femoris	NF	^*∗*^−2.04 (10.4)	^*∗*^−0.45 (9.74)	−0.00034 (0.003)	−0.00043 (0.004)	^*∗*^0.80 (2.36)	^*∗*^0.75 (2.33)	0.09 (0.82)	^*∗*^0.24 (0.78)	^*∗*^0.22 (0.80)
	PF	7.55 (8.23)	5.11 (6.46)	−0.00033 (0.0028)	−0.00049 (0.0037)	−0.70 (2.69)	−0.80 (2.30)	−0.22 (0.85)	−0.27 (0.92)	−0.34 (0.98)

Rectus femoris	NF	^*∗*^−4.67 (9.91)	^*∗*^−2.37 (6.67)	^*∗*^−0.00061 (0.0018)	^*∗*^−0.00066 (0.0024)	^*∗*^1.02 (2.60)	^*∗*^1.03 (2.64)	^*∗*^0.36 (1.11)	^*∗*^0.10 (1.02)	^*∗*^0.10 (1.13)
	PF	5.77 (10.22)	6.43 (8.56)	0.001388 (0.0020)	0.0017 (0.0024)	−2.29 (2.51)	−2.36 (2.61)	−0.75 (0.76)	−0.66 (1.01)	−0.74 (1.14)

Vastus lateralis	NF	^*∗*^−2.07 (8.45)	^*∗*^−2.15 (9.00)	−0.00038 (0.0022)	−0.00054 (0.0029)	0.40 (2.74)	^*∗*^0.66 (2.66)	0.17 (0.94)	^*∗*^0.13 (0.87)	0.11 (0.88)
	PF	6.34 (7.85)	4.64 (7.58)	−0.00052 (0.0024)	−0.00064 (0.0031)	−0.59 (2.38)	−0.60 (2.41)	−0.21 (0.83)	−0.26 (0.85)	−0.25 (0.92)

Vastus medialis	NF	^*∗*^−0.75 (7.59)	^*∗*^−0.30 (7.42)	0.0001 (0.002)	0.0001 (0.003)	0.58 (2.74)	0.54 (3.17)	0.24 (1.02)	0.12 (0.96)	0.11 (0.95)
	PF	4.06 (4.81)	4.28 (5.77)	0.00022 (0.0024)	0.000246 (0.0032)	−0.330 (1.75)	−0.38 (1.73)	−0.10 (0.96)	−0.02 (0.66)	−0.01 (0.58)

^*∗*^The differences differ significantly tested using the *t*-test at *P* < 0.05.

**Table 5:** Classification results of prolonged fatigue based on the naïve Bayes method.

Parameter		Muscles			
Features	Performance	BF	RF	VL	VM
Time features (ΔMAV, ΔRMS)	Accuracy (%)	70	78	64	56
	Specificity (%)	100	84	83	97
	Precision (%)	0	67	36	0
	CVErr	0.31	0.25	0.43	0.44

Frequency features (Δ*F*_med_, Δ*F*_mean_)	Accuracy (%)	86	95	68	77
	Specificity (%)	88	94	89	83
	Precision (%)	79	96	36	69
	CVErr	0.15	0.04	0.39	0.23

Time and frequency features (ΔMAV, ΔRMS, Δ*F*_med_, Δ*F*_mean_)	Accuracy (%)	94	98	95	97
	Specificity (%)	97	100	100	97
	Precision (%)	86	96	88	96
	CVErr	0.06	0.01	0.07	0.02

Wavelet index features (ΔWIRM1551, ΔWIRM1M51, ΔWIRM1522, ΔWIRE51, ΔWIRW51)	Accuracy (%)	82	91	80	66
	Specificity (%)	85	93	78	71
	Precision (%)	77	89	84	58
	CVErr	0.18	0.09	0.23	0.38

Time, frequency, and wavelet index features (ΔMAV, ΔRMS, ΔFmed, ΔFmean, ΔWIRM1551, ΔWIRM1M51, ΔWIRM1522, ΔWIRE51, ΔWIRW51)	Accuracy	87	88	77	90
	Specificity	86	91	79	83
	Precision	89	85	80	100
	CVErr	0.16	0.15	0.23	0.2

## Data Availability

The surface electromyography physiology data used to support the findings of this study have not been made available because they involve the third-party right and participant privacy.

## Supplementary Materials

Supplementary Materials Table S1: schedule of the experiment. Table S2: Bruce Protocol treadmill test. Table S3: prolonged fatigue sign identification. Table S4: quadriceps muscle movement based on the flexed and extended knee. Appendix S1: PAR-Q and You. Appendix S2: training log data collection form. Appendix S3: 24-hour history training distress questionnaire. Figure S1: flowchart of the experimental procedure and data collection. Figure S2: schematic circuit of the surface EMG data acquisition system.
