# Supplementary material for: A New Approach to Noninvasive-Prolonged Fatigue Identification Based on Surface EMG Time-Frequency and Wavelet Features
Source: J Healthc Eng. 2023 Jan 30;2023:1951165. doi: 10.1155/2023/1951165 (PMC9902121; doi:10.1155/2023/1951165)

**SUPPLEMENTARY MATERIALS**

Table S1. Schedule of Experiment

| Day 1 | Day 2 | Day 3 | Day 4 | Day 5 | Day 6 | Day 7 | Day 8 | Day 9 | Day 10 | Day 11 | Day 12 |
| --- | --- | --- | --- | --- | --- | --- | --- | --- | --- | --- | --- |
| PHASE I: FAMILIARIZATION WEEK | | | | | REST | | PHASE II: INTENSIVE TRAINING | | | | |
| ✓ |  | ✓ |  | ✓ |  |  | ✓ | ✓ | ✓ | ✓ | ✓ |

Table S2. Bruce Protocol Treadmill Test

| Stage | Speed (km/h) | Incline (° grade) | Duration (min) |
| --- | --- | --- | --- |
| 1 | 2.7 | 10 | 3 |
| 2 | 4.0 | 12 | 3 |
| 3 | 5.4 | 14 | 3 |
| 4 | 6.7 | 16 | 3 |
| 5 | 8.0 | 18 | 3 |
| 6 | 8.8 | 20 | 3 |
| 7 | 9.6 | 22 | 3 |

| **Tools** | **Prolonged Fatigue Signs** | **Identification** |
| --- | --- | --- |
| **Training Log** | Performance Decrement | Endurance time previous workout better |
|  | Restlessness | HR>100 before running |
|  | Hypertension | BP>140/90 before running |
| **24 Hours Training Distress Questionnaire** [24] | Sleeping Disturbance | The different time duration between before and during intensive training |
|  | Psychological Disturbance | Psychological score >14 |
|  | Muscle Soreness | Soreness scale  (Scale 4 - Tender but not sore to scale 7 – Very very sore) |
| **Interview** | Unexplained lethargy | Feel lethargy before running |
| **Borg Scale CR10** [30] | The difficulty level of exercise increase | Increasing of scale |

Table S3. Prolonged fatigue signs identification

Table S4 Quadriceps muscle movement based on flexed and extended knee

| Knee Movement | Quadriceps Muscles Response/ Surface EMG Signal |
| --- | --- |
| Flexed Knee  (Konrad, 2005)  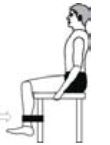 | Relax / Baseline |
| Extended Knee (Konrad, 2005)  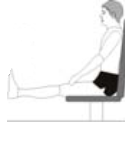 | Active / Contraction |

APPENDIX S1: Par-Q and You

**
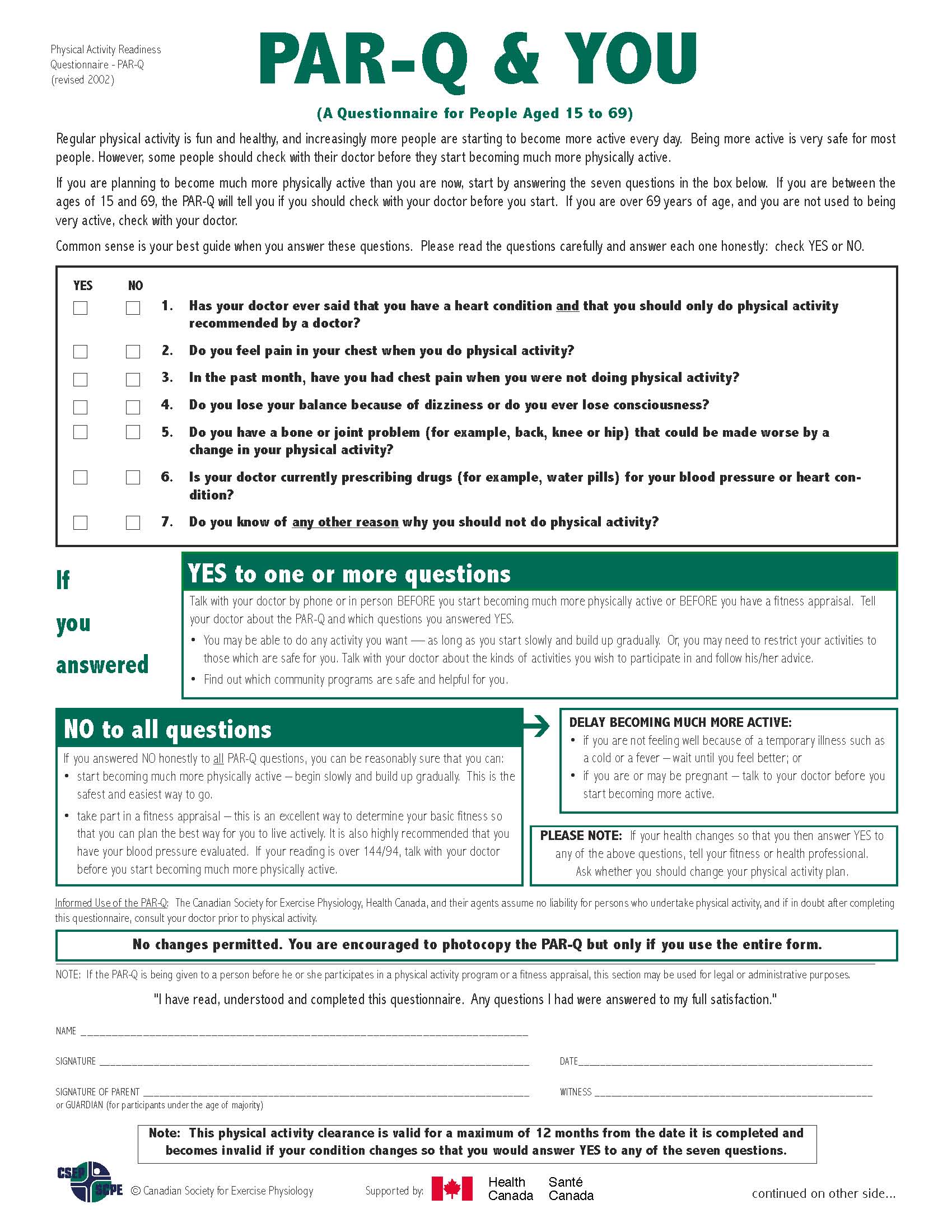
**

**
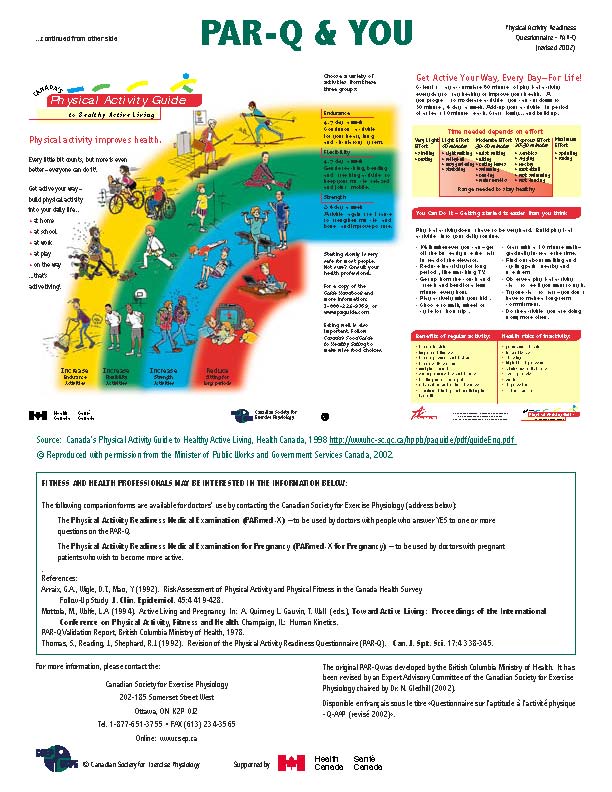
**

APPENDIX S2: Training Log Data Collection Form


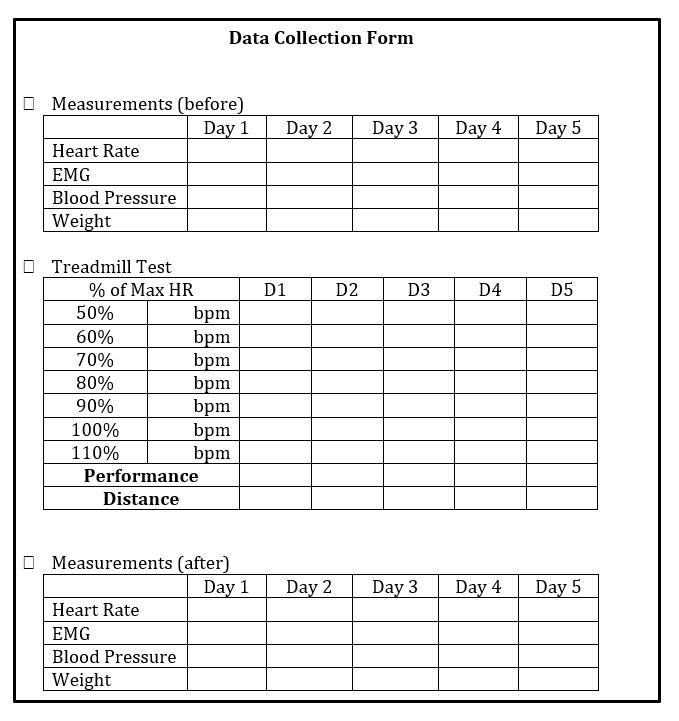


APPENDIX S3: 24-Hours History Training Distress Questionnaire

Questions:

1. How do you feel today? (Circle your response)
2. Very, very good
3. Very good
4. Good
5. Average
6. Bad
7. Very bad
8. Very, very bad
9. How many hours did you sleep last night?

_______________________________________________

1. How many hours do you normally get?

______________________________________________

1. Have you been sick the past week?
   ____________________________________________
2. How would you rate yesterday’s workout? (circle your response)
3. Very, very easy
4. Very easy
5. Easy
6. Average
7. Bad
8. Very bad
9. Very, very bad
10. How do your muscles feel? (legs) (circle your response)

1. Very, very good

2. Very good

3. Good

4. Tender, but not sore

5. Sore

6. Very sore

7. Very, very sore

Please respond to the following items as to how you have been feeling the last week, including today:

|  | Not at all | A little | Moderately | Quite a bit | Extremely |
| --- | --- | --- | --- | --- | --- |
| Friendly | 0 | 1 | 2 | 3 | 4 |
| Worthless | 0 | 1 | 2 | 3 | 4 |
| Miserable | 0 | 1 | 2 | 3 | 4 |
| Helpful | 0 | 1 | 2 | 3 | 4 |
| Bad-tempered | 0 | 1 | 2 | 3 | 4 |
| Guilty | 0 | 1 | 2 | 3 | 4 |
| Unworthy | 0 | 1 | 2 | 3 | 4 |
| Peeved | 0 | 1 | 2 | 3 | 4 |
| Cheerful | 0 | 1 | 2 | 3 | 4 |
| Sad | 0 | 1 | 2 | 3 | 4 |

NO

YES

NO

YES

Finished Running

MEASUREMENT

(Surface EMG, Heart Rate, Blood Pressure, Borg Scale)

Improve Performance?

Day 5?

Stretching

Bruce Protocol Treadmill Test

(Measure endurance time, %HRmax, observe Fatigue Symptoms 2)

Briefing on Fatigue Symptoms

MEASUREMENT

(Surface EMG, Heart Rate, Blood Pressure, Short Interview, Questionnaire)

Fatigue Symptoms 1?

YES

**Figure S1. Flow chart of the experimental procedure and data collection**

**Appendix S2: Schematic Circuit of Surface EMG Data Acquisition System**

1. **Power Supply Circuit**


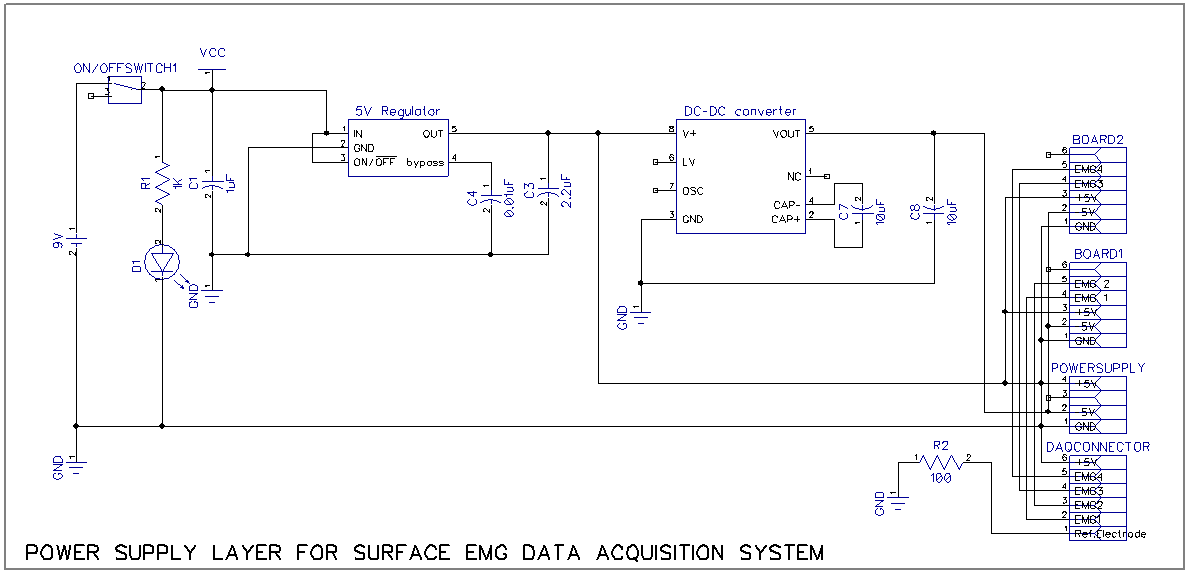


1. **Data Acquisition Circuit**


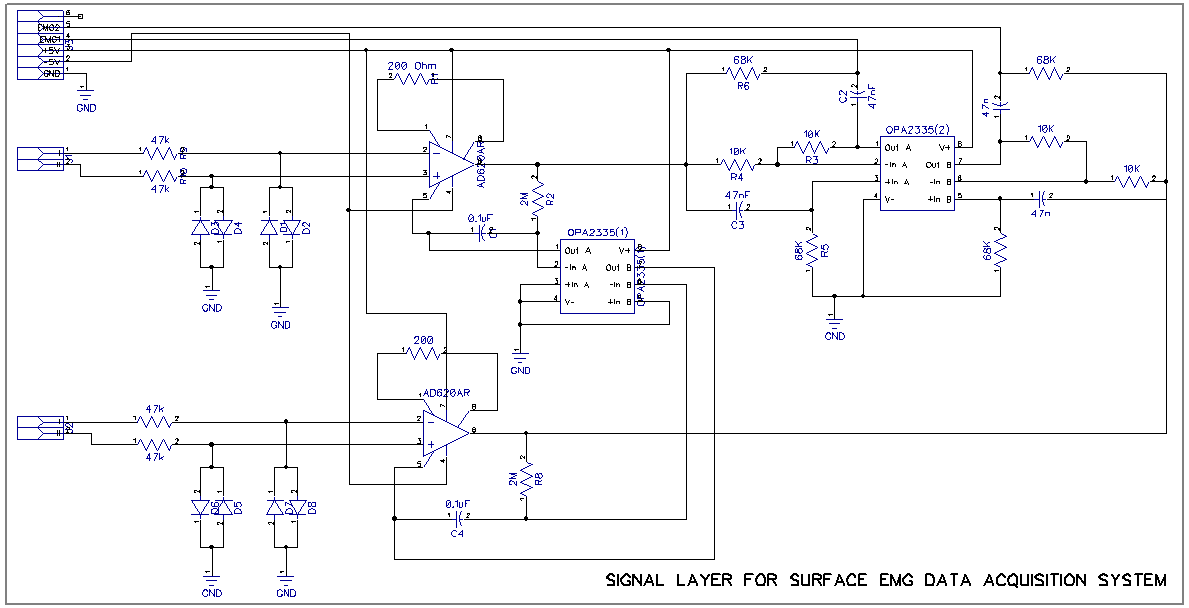

Supplement: Supplementary Materials — Table S1: schedule of the experiment. Table S2: Bruce Protocol treadmill test. Table S3: prolonged fatigue sign identification. Table S4: quadriceps muscle movement based on the flexed and extended knee. Appendix S1: PAR-Q and You. Appendix S2: training log data collection form. Appendix S3: 24-hour history training distress questionnaire. Figure S1: flowchart of the experimental procedure and data collection. Figure S2: schematic circuit of the surface EMG data acquisition system. [file 1951165.f1.docx]
